# Introducing a digital emergency obstetric and newborn care register for indoor obstetric patient management: An implementation research in selected public health care facilities of Bangladesh

**DOI:** 10.7189/jogh.14.04075

**Published:** 2024-05-10

**Authors:** Sabrina Jabeen, Mahiur Rahman, Abu Bakkar Siddique, Mehedi Hasan, Rubaiya Matin, Qazi Sadeq-ur Rahman, Tanvir Hossain AKM, Azizul Alim, Nuzhat Nadia, Mustufa Mahmud, Jahurul Islam, Muhammad Shariful Islam, Mohammad Sabbir Haider, Farhana Dewan, Ferdousi Begum, Uchchash Barua, Mohammad Toriqul Anam, Abirul Islam, Khandaker Sabit Bin Razzak, Shafiqul Ameen, Aniqa Tasnim Hossain, Quamrun Nahar, Anisuddin Ahmed, Shams El Arifeen, Ahmed Ehsanur Rahman

**Affiliations:** 1 International Centre for Diarrhoeal Disease Research, Dhaka, Bangladesh; 2 Directorate General of Health Services, Ministry of Health and Family Welfare, Government of the People’s Republic of Bangladesh, Dhaka, Bangladesh; 3 Obstetrical and Gynaecological Society of Bangladesh, Dhaka, Bangladesh

## Abstract

**Background:**

Digital health records have emerged as vital tools for improving health care delivery and patient data management. Acknowledging the gaps in data recording by a paper-based register, the emergency obstetric and newborn care (EmONC) register used in the labour ward was digitised. In this study, we aimed to assess the implementation outcome of the digital register in selected public health care facilities in Bangladesh.

**Methods:**

Extensive collaboration with stakeholders facilitated the development of an android-based electronic register from the paper-based register in the labour rooms of the selected district and sub-district level public health facilities of Bangladesh. We conducted a study to assess the implementation outcome of introducing the digital EmONC register in the labour ward.

**Results:**

The digital register demonstrated high usability with a score of 83.7 according to the system usability scale, and health care providers found it highly acceptable, with an average score exceeding 95% using the technology acceptance model. The adoption rate reached an impressive 98% (95% confidence interval (CI) = 98–99), and fidelity stood at 90% (95% CI = 88–91) in the digital register, encompassing more than 80% of data elements. Notably, fidelity increased significantly over the implementation period of six months. The digital system proved a high utility rate of 89% (95% CI = 88–91), and all outcome variables exceeded the predefined benchmark.

**Conclusions:**

The implementation outcome assessment underscores the potential of the digital register to enhance maternal and newborn health care in Bangladesh. Its user-friendliness, improved data completeness, and high adoption rates indicate its capacity to streamline health care data management and improve the quality of care.

Emergency obstetric and newborn care (EmONC) constitutes a specialised component of maternal health in Bangladesh. It aims to address complications during pregnancy, childbirth, and the postpartum phase to ensure women’s well-being [1]. For individual-level tracking of this service, an EmONC register (e-register) has been introduced, manually maintained by midwives and labour room staff, to document the delivery-related data of service users [1].

EmONC register is currently paper-based, where health care providers record data following deliveries. However, the use of paper-based registers presents several challenges. Shift changes during labour can lead to the risk of forgetting to record important information, potentially resulting in incomplete data. Additionally, delayed data entry further compounds this issue and increases the likelihood of losing relevant and critical information if not promptly completed [2]. Moreover, at the end of the month, the data from the register is compiled and plugged in manually to the central District Health Information Software2. Transferring and handling the same data through multiple platforms and individuals introduces several opportunities for human error [3–5]. Consequently, for retrospective data analysis, only monthly data are available, and real-time data checking requires the physical registers to be transported, potentially accelerating the preservation challenge of the register and delays in accessing critical health care information [6,7].

In response to the limitations of paper-based registers, a digitisation effort led to creating a digital EmONC e-register with a target to improve the data quality and real-time access to health-related data. Nevertheless, digital health records represent promising tools for routine health care, and the analysis of electronic health records has emerged as an increasingly popular approach for investigating real-world patient data [8,9]. A fundamental element of digital health revolves around the collection and utilisation of electronic health data to enhance health and overall well-being [10]. Though digital systems may vary in features and implementation across diverse settings, they serve the uniform purpose of capturing, storing, and tracking patient information [11]. As countries worldwide embrace the digital revolution in health care, it becomes imperative to delve into both the benefits and challenges accompanying this transformative trend. Digitisation initiatives hold particular promise in resource-constrained settings like Bangladesh, where access to real-time and quality health data are limited.

What distinguishes this e-register are its distinct features, including immediate data accessibility, decision support functionalities, mandatory input fields, error-checking mechanisms, and timely reminders. Furthermore, its integration with a dedicated dashboard fulfils the crucial role of continuous monitoring, contributing significantly to the overall efficiency of health care management. We conducted implementation research because we did not have any prior information regarding the implementation challenges and outcome of transitioning a register from a traditional paper-based format to a digital platform. In this paper, we aimed to assess the implementation outcome of this digital EmONC register, considering the World Health Organization (WHO) implementation research variables [12].

## METHODS

### Study design

We conducted implementation research based on the WHO-recommended implementation research variables to assess the outcome during the design, development, and demonstration of an Android-based electronic register for EmONC services [12]. The transformation of this paper into a digital format involved extensive collaboration with various stakeholders [13]. We identified stakeholders through a power-mapping matrix exercise of those playing pivotal roles in maternal health services in Bangladesh. Throughout the process, we followed the four-step stakeholder engagement process (identification, sensitisation, involvement, and engagement) [13]. The stakeholders involved the Maternal Health Programme, National Newborn Health Programme and Integrated Management of Childhood Illness (IMCI) of the Directorate General Health Services of the Government of Bangladesh, WHO, United Nations International Children’s Emergency Fund, United Nations Population Fund, Ipas, Save the Children, and Obstetrical and Gynaecological Society of Bangladesh. The international research organisation icddr,b (formerly known as the International Centre for Diarrhoeal Diseases Research), located in Bangladesh, played a crucial role by offering technical expertise and assistance throughout the process, including developing, facilitating, and evaluating the viability of the digital EmONC register and overall collaboration among the stakeholders. The details of the stakeholder engagement process have been described in a separate paper.

### Study duration and setting

We conducted research from November 2021 to February 2023, in the Kushtia and Dinajpur districts of Bangladesh. The selection of Kushtia and Dinajpur Districts for this study was meticulously considered by stakeholders while factoring in geographical and socioeconomic variables. In addition, the health indicators of these districts show an upright pattern, which is also a positive factor for selecting the districts. Kushtia is situated in the southwestern region of the country, approximately 200 km away from Dhaka, while Dinajpur is in the northern part of the country, around 300 km north of Dhaka. For this research, the Maternal health program, in collaboration with the district health manager (civil surgeon) and sub-district health managers (Upazila Health and Family Planning officers), selected one district hospital (providing secondary-referral services) and one sub-district hospital (offering primary-referral services) from each of these districts as the implementation facilities (Figure S1 and Table S1 in the **Online Supplementary Document**).

### Development of the e-register application

The digital EmONC register was created with active involvement from the Maternal Health Programme, National Newborn Health Programme and IMCI, development partners and professional bodies dedicated to work in maternal health. Before developing the application, we visualised the existing data recording system that needed to be digitised. This process involved a thorough understanding of the 50 variables found in the current paper-based EmONC register, which were categorised into five sections: patient registration, delivery-related information, essential newborn care, post-natal care, and discharge details. The application was designed to capture all 50 variables in a sequence similar to the paper-based register (Figure S2 in the **Online Supplementary Document**). It also featured decision prompts and error-checking options. Notably, once patient data was entered, the application prevented any editing after 24 hours to minimise the risk of recall errors. The EmONC application also includes guidance on patient care and providing maternal health resources to health care providers, specifically the Government of Bangladesh’s patient management guidelines and other relevant documents. Additionally, we incorporated a dashboard feature into the application, offering graphical visualisations of real-time facility data. The e-register application includes a companion web-based dashboard designed for facility managers to track their facility’s real-time performance [14].

The development process of the application comprised several vital steps. Initially, we organised sensitisation meetings and consultative workshops with the stakeholders to revamp the list of indicators and create an algorithm based on their feedback. Subsequently, we designed the user interface and arranged the dashboard layout with a focus on the user experience [15]. Following this, the application and dashboard were constructed, and the research physicians of the team initiated alpha testing. The alpha testing result guided adjustments to the application and the dashboard. Next, a beta version of the application was used to provide training and orientation to health care providers in the selected health care facilities for outcome assessment [16].

### District implementation model

The Maternal Health Programme, the National Newborn Health Programme, and IMCI collaborated with other stakeholders through stakeholder engagement to design and develop a district implementation model [13,17]. This approach adopted a health system strengthening perspective and consisted of the following four key components. At first, sensitisation of the district and sub-district level facility managers and the health care providers working in the labour room through workshops and involving them in planning and implementation. Second, capacity development of the health care providers working in the labour room and obstetric operation theatre. Third, the implementation is done by the distribution of tablet computers and the establishment of internet connections in the implementation facilities. Finally, follow-up support provided to the labour room service providers, routine monitoring and supportive supervision.

### Sensitisation and planning

In each district, we conducted a sensitisation workshop to inform and engage facility managers and health care providers in recognising the significance of transitioning from a paper-based data recording system to a digital one. Further, with this workshop, we aimed to outline the framework for the implementation plan. Subsequently, individual facility-level planning meetings were held involving facility managers, health care providers and their clinical supervisors. These meetings were focused on facility-specific microplanning and preparations for the introduction of digital EmONC registers in labour rooms and operation theatres.

### Capacity development

We conducted district-specific training sessions for frontline health care providers, including midwives, nurses with six months of midwifery training, and statisticians working in the selected facilities. 101 health care providers underwent training in six separate batches across both districts. These training sessions were facilitated by the research team and data management support team from icddr,b and included lectures, live demonstrations, hands-on practice sessions using tablet computers, mock tests, and feedback sessions. Additionally, a session was held to instruct participants on using the dashboard.

### Implementation of digital EmONC register

We began the implementation of the digital EmONC register in the chosen health facilities immediately after completing the training sessions in each district. Tablet computers were strategically positioned in the labour rooms and operation theatres as per prior discussions with the facility managers at the implementation facilities, and paper-based registers were withdrawn. icddr,b’s data management support team set up the facility system and activated the web-based dashboard on the facility managers’ computers.

### Follow-up support

Following the implementation of the e-register, icddr,b’s data management support team offered one week of hands-on support and active guidance in these implementing facilities. This support involved real-time troubleshooting and reorienting facility managers, health care providers, and statisticians. Subsequently, the support team regularly visited the facilities during the implementation phase to provide troubleshooting and active guidance. District and sub-district facility managers routinely used the dashboard to monitor activities in the labour room and provided feedback accordingly. Senior obstetricians closely monitored the data recording on the newly established platform during this period.

### Study participants

Our primary study population included front-line service providers (caregivers) who conduct deliveries, such as doctors, nurses, and midwives, as well as policymakers, facility managers, and statisticians. The secondary study population included care receivers (mothers who have delivered their child in any of the four implementing facilities).

### Sample size

We calculated the sample size for each WHO implementation outcome variable (**Table 1**). We enrolled 32 participants for usability and acceptability. For adoption, fidelity, and utility, we aimed to assess 414 delivery data.

**Table 1:** WHO’s implementation outcome variables, research questions and benchmarks finalised through stakeholder consultation

WHO’s framework	Research question	Proposed indicator	Benchmark (%)
Usability	Is the e-register usable for the health care providers?	Average score using SUS	68
Acceptability	Is the e-register acceptable to the health care providers?	Average score using the components of the TAM	>80
Adoption	Actual use: Do the providers use e-register register?	The proportion of deliveries recorded by digital EmONC register	>90
Fidelity	Completeness: Do the providers input information completely in the e-register?	The proportion of livebirths with more than 80% data elements recorded (80% close to actual completeness)	>75
Utility	Quality of care: Does the e-register ensure quality of care?	The proportion of livebirths with a valid record on AMTSL, birth weight and CHX application	80

AMTSL – active management of the third stage of labour, CHX – chlorhexidine, EmONC – emergency obstetric and newborn care, SUS – system usability scale, TAM – technology acceptance model

### Data collection

We employed quantitative data collection methods for this assessment. We utilised the data entered into the e-register for evaluation purposes. Additionally, we extracted the information from the paper-based EmONC registers at the selected four facilities, which covered three months (April–June). Simultaneously, we examined District Health Information Software2 delivery records for the same time duration to assess the utilisation of the paper-based register. To obtain the data on patient admission in the labour ward during implementation, data collectors were deployed in shifts, around the clock, seven days a week, to record the total number of patients admitted to the labour ward in an individual record, enabling the calculation of the overall number of deliveries in these facilities.

### Data analysis

To assess the usability of the e-register, we used the system usability scale score, where the cut-off score is considered to be equal to or more than 68% [18]. We also calculated the acceptability by average scores of participants for the five components of the technology acceptance model, which include perceived usefulness, perceived ease of use, attitude toward using, behavioural intention, intention to use and actual use [19]. We used descriptive statistics to present the findings related to the adoption, fidelity, and utility of the e-register. The uncertainty rate (UR) was calculated using a 95% confidence interval for outcome variables, which quantified the range within which the true values of outcome variables were likely to fall. The result was also disaggregated by month, district, and facility type.

To measure fidelity, we identified a set of ten variables (receiving antenatal care, active management of the third stage of labour, provider who conducted the delivery, any other operation required, mother’s postnatal care, newborn’s postnatal care, postpartum family planning, birthweight, use of chlorhexidine and maternal complication), all of which required completion to ensure data records. Additionally, to assess the utility aspect, specific variables targeting newborns and mothers were pinpointed in consultation with stakeholders and through desk review [17,20]. In the case of newborns, the focus was on the recording of birth weight and the use of chlorhexidine and for mothers, the emphasis was placed on active management of the third stage of labour (AMTSL). For quantitative data analysis, we used the statistical software STATA, version 15.0 (StataCorp LLC, TX, USA).

## RESULTS

**Figure 1:**
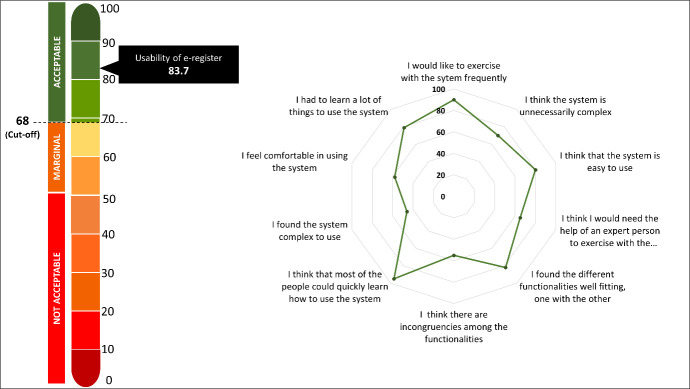
Usability score of digital EmONC register using system usability scale (n = 32).

**Figure 2:**
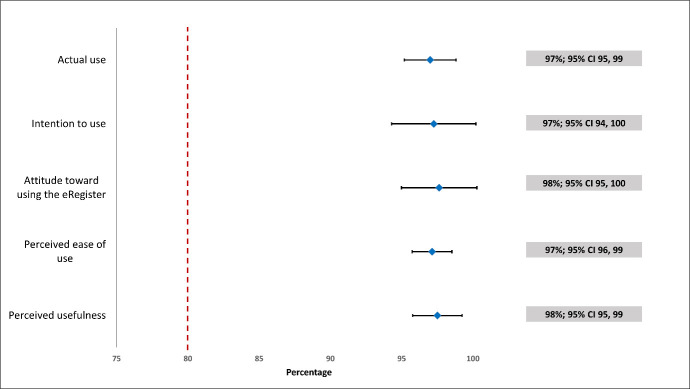
The average score of acceptability of the e-register in the components of the technology acceptance model (n = 32).

The measured usability of the e-register using the average score of the system usability scale was 87.3 with a cut-off value of 68 [18] (**Figure 1**). All five components of the technology acceptance model received an average score exceeding 95% (**Figure 2**). Out of the 1644 deliveries recorded in the e-register, 1568 resulted in live births. Similarly, out of the 1235 deliveries recorded in the paper-based EmONC register, 1140 were live births (**Table 2**).

**Table 2:** Total number of delivery data collected from the different data sources in three months

Data source	Recorded number of deliveries	Duration
Paper-based EmONC register		
*Total deliveries in the facility*	1235	April–June 2022
*Total live births in the facility*	1140	
District Health Information Software2		
*Total deliveries in the facility*	1414	April–June 2022
Digital EmONC register		
*Total deliveries recorded in the facility*	1644	November–December 2022; January 2023
*Total live births recorded in the facility*	1568	
Data collectors register		
*Total deliveries in the facility*	1675	November–December 2022; January 2023

EmONC – emergency obstetric and newborn care

**Figure 3:**
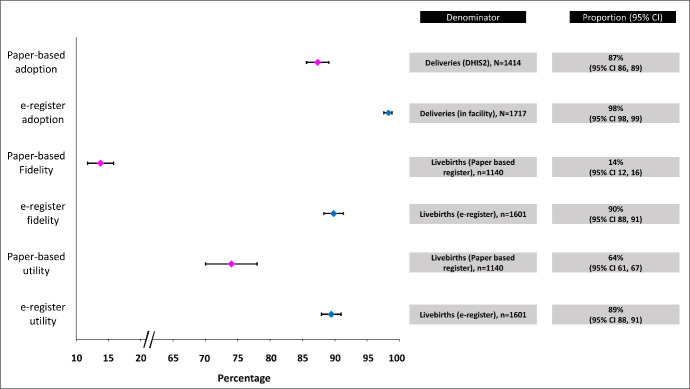
Comparison of the proportion of adoption, fidelity and utility (WHO implementation research outcome variables) recorded by the paper-based register and e-register.

During the implementation period, the adoption rate of the digital register was 98%. In terms of fidelity, the electronic register achieved a rate of 90%, while the paper-based register displayed a much lower fidelity rate of 14%. Furthermore, the utility of the digital EmONC register was 89%, which exceeded the utility rate of the paper-based register, which stood at 64% (**Figure 3**).

**Figure 4:**
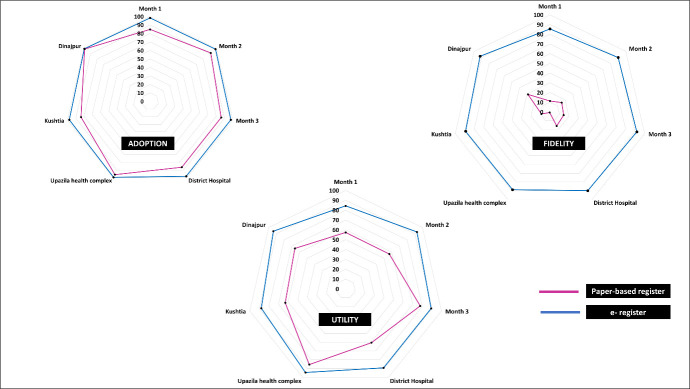
Comparison of the adoption, fidelity and utility (WHO implementation research outcome variables) by month, facility, and district recorded by the paper-based register and e-register.

The adoption proportion consistently exceeded 95% each month, with the peak occurring in the middle of the implementation period. Fidelity in using the e-register increased progressively as the implementation duration advanced, reaching a remarkable peak of 92% (UR = 90–94) in the last month. This rising trend was evident across all facility types. The utility of the electronic register was most pronounced in the second month, which was 93% (UR = 91–95) (**Figure 4**, Table S2 in the **Online Supplementary Document**).

**Figure 5:**
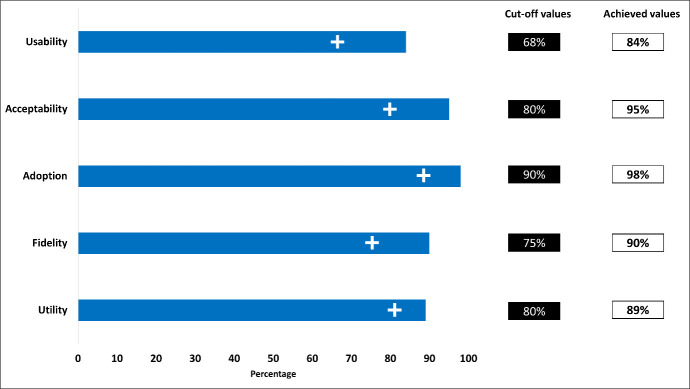
The achieved values of the implementation outcome variables in comparison to the cut-off values for assessing the e-register.

**Figure 6:**
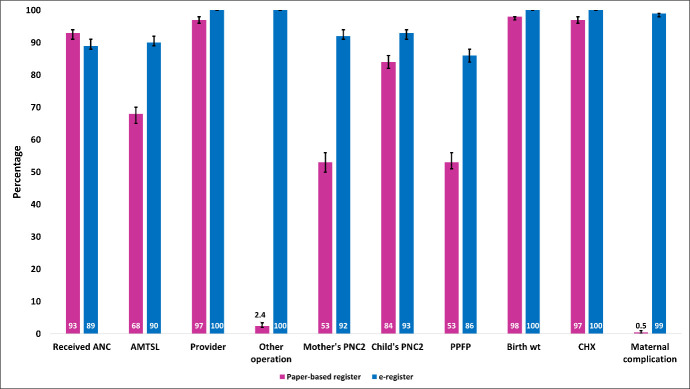
The proportion of variables used to measure the fidelity and utility of paper-based (n = 1140) registers and e-registers (n = 1568) registers.

Regarding the proportion of the WHO variables that achieved the benchmark, it was evident that all the variables successfully exceeded the benchmark value. For the distribution of the variables used for assessing the fidelity and utility of the paper-based register and the digital EmONC register, the proportion of all the variables increased in the case of recording to the digital register, which was more than 90% except for antenatal care, active management of the third stage of labour, and postpartum family planning (**Figure 5** and **Figure 6**).

## DISCUSSION

With this research, we aim to assess the outcome of implementing the digital EmONC registers (e-register) with the outcome variables of the WHO implementation research [12]. The health care providers who used the e-register expressed a strong inclination towards its usability and acceptability, acknowledging the minor technical glitches during their use. Furthermore, the adoption, fidelity, and utility of the digital register remarkably exceeded those of the paper-based register, surpassing the predefined benchmarks.

The e-register’s usability indicates higher efficiency, effectiveness and satisfaction of the user [21]. The digital EmONC register was designed with a strong emphasis on user experience, considering feedback from health care providers and stakeholders. It effectively transformed all the variables from paper-based records into an easily understandable digital format, simplifying the learning and use for health care providers. Given the diverse social, cultural, and economic barriers they encounter, nurses and midwives, who are central to using this and crucial in women’s informed health decision-making, face difficulties in keeping up with technological advancements [22,23]. Despite these challenges, the straightforward and user-friendly design of the e-register has delivered remarkable results. Similarly, we observed strong indications of enhanced usability in an electronic office system, which attained a usability score of 78.45; similarly, electronic health records developed in a health care facility in East Java scored 77.14 on the system usability scale [18,24]. In contrast to our results, a survey was conducted in an emergency medicine department in the United Kingdom to measure the usability of the electronic health record system using the technology acceptance model. Unfortunately, none of the electronic health recording systems met the internationally accepted standard of acceptability. The average score was 53 (interquartile range = 35–68) [25].

Perceived usefulness, ease of use, attitude towards using the application, intention to use and actual use of the digital register among the health care providers scored more than 95%. Looking for evidence of a study testing an electronic medical report in a hospital in Malaysia, we found the proportion of perceived usefulness, perceived ease of use, and intention to use was more than 60% [26]. Several external variables, including one’s profession, general experience, familiarity with similar technology, computer skills, and specific technology training, have been recognised as influential factors affecting the perceived ease of use and usefulness of a technology [19,27]. Although most health facilities in Bangladesh still rely on manual paper-based registers for data recording, the monthly requirement to submit data into the computer-based server District Health Information Software2 is mandatory for all public health care facilities [3]. Enhanced data security and a user-friendly interface can potentially increase the perceived usefulness, as indicated by a study that identified compatibility with the system, its security, and reliability as external factors affecting users' perception of ease of use and usefulness [28]. Conversely, contrary findings also suggest that certain external factors like gender, experience, and profession do not influence how users perceive the ease of use or usefulness of technology [29].

We found that the digital register could record more data than the paper-based register. Previously, a common practice in health care facilities involved using separate registers in the labour room and the operating theatre. This led to inadvertent omissions of numerous cases during the monthly collation process. However, a solution was found by deploying multiple devices with the same digital register application in these areas, allowing them to synchronise within all the data entry points. This reduced the burden on health care providers, eliminated the need for multiple entries for a single patient, and effectively prevented data loss. Using interconnected data recording points to create a peer-to-peer network has successfully decreased documentation loss, improved shift-to-shift reporting, and eliminated the need for multiple entries for a single patient. This approach has been highly effective in mitigating data loss [30,31]. Multiple studies have provided evidence that digital registers can accommodate a higher volume of patients compared to paper-based registers [32]. This increased patient recording capacity is particularly evident in digital registers when dealing with high patient loads [33]. In contrast to the findings, establishing an electronic health recording system in the ophthalmology department of a health care facility showed lower adoption rates than before [34]. Also, a study in Ethiopia showed only a 52.8% (UR = 47.9–56.6) adoption rate of electronic health record systems [35].

The peak of adoption occurred during the second month of implementation. This can be attributed to the novelty of the e-register in the first month, which required health care providers to become acquainted with it. Over time, their familiarity with the system grew. Moreover, during this initial period, the research team provided active support and guidance for data entry. However, in the third month of implementation, the focus shifted towards concluding fieldwork. As a result, research staff were occupied with wrapping up activities, and there was a reduction in the level of facilitation. This decrease in support had a noticeable impact on the adoption rate during that month. One study in Eastern Province, Saudi Arabia, found that the absence of continuous support created barriers for health care staff in data handling, while another study indicated that retaining support resulted in an improved data recording system [36,37].

Our implementation research indicated that the e-register could document over 96% of deliveries completely, in contrast to the paper-based register. Inside the digital application, decision prompts were in place, offering health care providers reminders regarding the subsequent steps in patient care and assisting in data entry for these actions. This functionality effectively prevented providers from neglecting to fill in fields and motivated them to provide comprehensive information. Including such warnings, reminders, and decision-guidance features in electronic data recording systems gave the e-register a distinct advantage over traditional paper-based data recording systems [38,39]. The dashboard feature of the e-register showcased the level of data completeness at various cutoff points, a further initiative aimed at enhancing data quality, as immediate data visibility works to reduce human error [40]. Similar to these findings, a study conducted in Seoul, South Korea, demonstrated improved data completeness in electronic recording systems, while another study reported that electronic systems significantly outperformed paper-based systems in recording comprehensive eye examination findings [41,42]. Contrary to our findings, a Northwest Ethiopian study presented evidence suggesting nearly equivalent data completeness between electronic medical records and paper-based records [43].

The digital EmONC register demonstrated a steady rise in data completeness, reaching its peak during the third month of implementation. Real-time display of data to facility managers played a crucial role as a motivating factor for ensuring meticulous data entry. Feedback and peer monitoring are highly effective methods for continually enhancing and maintaining data quality [44]. It is important to recognise that becoming accustomed to a complex interface requires time, and as efficiency improves over time, so does the quality of the data [45]. One study’s findings from an urban hospital in West Tennessee indicated a significant enhancement in data quality after consistent and regular usage of the data recording system [37].

Digital EmONC register demonstrated an overall utility rate of 89%. Similarly, we found evidence of the 87% utility of an electronic recording system in a cancer hospital [46]. It was observed that the indicator for maternal health, active management of the third stage of labour (AMTSL), was recorded less frequently (90%) when compared to the indicators for newborn health, which included birth weight (100%) and the use of chlorhexidine (99%). Based on the Bangladesh Demographic Health Survey, Bangladesh has made significant progress toward achieving the sustainable development goal of reducing newborn and child mortality [47]. However, in contrast to the rapid decline observed in the first decade of this millennium, with an average annual reduction rate of 5%, the reduction rate of maternal mortality has slowed down considerably since 2010, now standing at only 2.1% [48–50]. Currently, haemorrhage remains the leading cause of maternal mortality in Bangladesh [49,50]. This disparity can result from the generic low prioritisation of rights and needs of women and inadequately trained maternal health workers, as reported by WHO [51]. There is substantial supporting evidence in the Bangladesh Health Facility Survey report indicating a shortage of adequately trained health care providers [52]. Active management of the third stage of labour (AMTSL) is a critical practice recommended by the WHO for preventing haemorrhage-related maternal deaths [53]. Regrettably, the data reveals a decline in training – in 2017, 41% of providers reported having received AMTSL training at some point, and by 2022, this figure had decreased to 32% [47,54]. The decrease in AMTSL training was also evident in the e-register, showing its impact through a reduction in the application of AMTSL practices.

The implementation outcome variable testing of the digital EmONC register application served as a valuable blueprint for a broader implementation. Having the paper-based register in place provided clear guidance for selecting variables, structuring a variable matrix, and developing algorithms. The collaborative effort of our research team and data management service team, with expertise in both technology and medical science, proved essential in addressing challenges arising from the intersection of these domains. Though the sample size of the study is seemingly small, we consider it adequate as the calculation was based on assuming a maximum variance of 50%, given the absence of relevant estimates in existing publications. However, the study team collected data from 1644 deliveries during the study period, which exceeds the calculated sample size of 414. The significance of this study becomes more pronounced as Bangladesh embarks on the doorway of a wider electronic health recording system. Such research is crucial for policymakers, providing evidence-based insights to guide decision-making.

This study has several limitations. First, the study sites were not chosen randomly. The sites were selected based on the consensus of the stakeholders, which was mostly dependent on the status of existing health indicators and facility setup. Second, the fact that member physicians from the research team tested the application could introduce reporting bias. To address this potential bias, we also involved physicians from other research teams during the alpha testing of the e-register. Third, we have only used WHO implementation variables to assess the outcome. However, there could be more that were not addressed. Lastly, we could collect data only for three months due to time limitations. However, having these constraints, we could collect more cases than our calculated sample size.

## CONCLUSIONS

The implementation outcome of the digital EmONC register yielded encouraging results. Along with the dashboard, this was a complete package for data collection and monitoring. Coupled with the dashboard, this constitutes a comprehensive solution for data collection and monitoring. In the era of digitalisation, transitioning to a digital system offers significant advantages, including improved data quality, streamlined record-keeping processes, and an increased capacity to handle a larger patient volume. These outcomes underscore the considerable potential of the digital register to substantially enhance maternal and newborn health care practices in Bangladesh and other lower-middle-income countries. However, using what has been learned from this study, the government of Bangladesh can scale up this digital register, and this large-scale expansion requires implementation research on its effectiveness. Furthermore, the outcomes of this study could potentially set a global example worth emulating in the context of electronic health systems.

## Additional material


Online Supplementary Document
