# Supplementary material for: Introducing a digital emergency obstetric and newborn care register for indoor obstetric patient management: An implementation research in selected public health care facilities of Bangladesh
Source: J Glob Health. 2024 May 10;14:04075. doi: 10.7189/jogh.14.04075 (PMC11082830; doi:10.7189/jogh.14.04075)
Supplement: Online Supplementary Document [file jogh-14-04075-s001.pdf]

Online Supplementary material

Figure S1: Study site

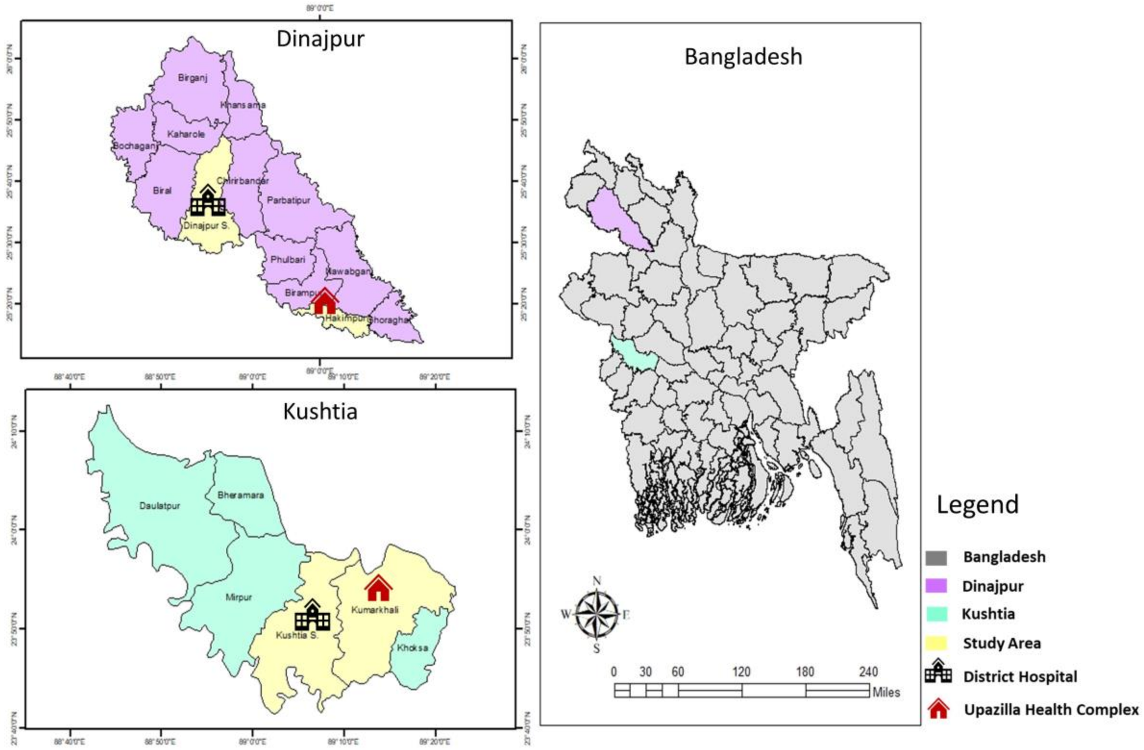

Figure S2: App wireframe

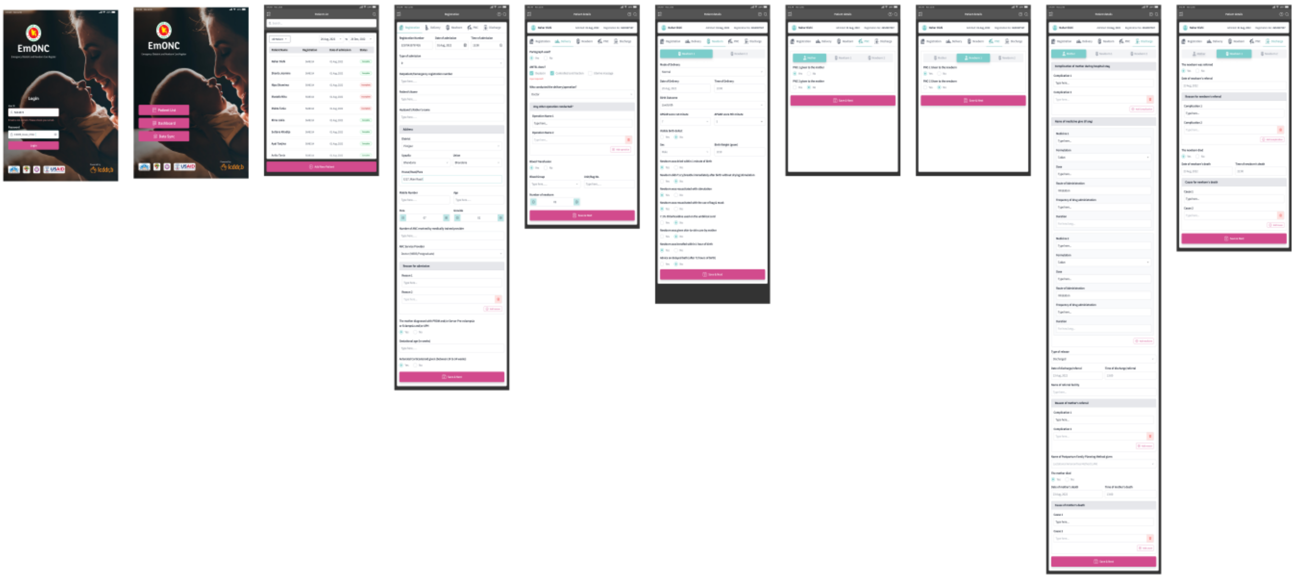

Supplementary material Table S1: Type, staffing pattern, service availability and EmONC service utilisation of health facilities.

Name	Type of Health Facility	Availability					Utilisation											
							NVD			Caesarian Section			Forceps/Vacuum/Breech Delivery			Still Births		
		Indoor service	Emergency service	Outdoor service	EmONC service	Separate Gynae & Obs ward	2021	2022	2023	2021	2022	2023	2021	2022	2023	2021	2022	2023
Kushtia DH	District Hospital	Yes, 250-bedded	Available	Available	Available	Available	1491	2012	946	1290	1972	943	11	26	19	154	224	115
Kumarkhali UHC	Upazila Health Complex	Yes, 50-bedded	Available	Available	Available	Available	385	454	200	33	45	35	1	-	-	9	4	7
Dinajpur DH	District Hospital	Yes, 250-bedded	Available	Available	Available	Available	700	725	282	736	677	279	4	2	1	23	20	10
Hakimpur UHC	Upazila Health Complex	Yes, 50-bedded	Available	Available	Available	Available	208	244	89	-	-	-	1	3	-	2	5	1

Supplementary material Table S2: Detailed distribution of the indicators in the Utility variable among all the live births in paper-based (N=1140) and digital register (N=1568)

	Birthweight				CHX				AMTSL			
	Paper-based register		Digital EmONC register		Paper-based register		Digital EmONC register		Paper-based register		Digital EmONC register	
	n	%	n	%	n	%	n	%	n	%	n	%
**Month**												
Month 1	371	98	336	100	368	97	337	100	235	62	285	85
Month 2	362	97	546	100	359	96	545	100	223	60	509	93
Month 3	379	98	684	100	376	97	675	99	313	81	624	91
**Facility**												
District Hospital	956	98	1405	99	949	97	1414	100	618	63	1274	90
Upazila Health Complex	156	96	152	100	154	95	152	100	153	94	144	95
**District**												
Kushtia	833	98	1170	99	833	98	1178	100	556	65	1052	89
Dinajpur	279	96	387	98	270	93	388	100	215	74	366	94
